# Inhibition of the TGFβ Pathway Enhances Retinal Regeneration in Adult Zebrafish

**DOI:** 10.1371/journal.pone.0167073

**Published:** 2016-11-23

**Authors:** Christoph Tappeiner, Ellinor Maurer, Pauline Sallin, Thomas Bise, Volker Enzmann, Markus Tschopp

**Affiliations:** 1 Department of Ophthalmology, Bern University Hospital, Inselspital, University of Bern, Bern, Switzerland; 2 Department of Biology, University of Fribourg, Fribourg, Switzerland; 3 Department of Ophthalmology, University Hospital of Basel, Basel, Switzerland; Wayne State University School of Medicine, UNITED STATES

## Abstract

In contrast to the mammalian retina, the zebrafish retina exhibits the potential for lifelong retinal neurogenesis and regeneration even after severe damage. Previous studies have shown that the transforming growth factor beta (TGFβ) signaling pathway is activated during the regeneration of different tissues in the zebrafish and is needed for regeneration in the heart and the fin. In this study, we have investigated the role of the TGFβ pathway in the N-methyl-N-nitrosourea (MNU)-induced chemical model of rod photoreceptor de- and regeneration in adult zebrafish. Immunohistochemical staining for phosphorylated Smad3 was elevated during retinal regeneration, and phosphorylated Smad3 co-localized with proliferating cell nuclear antigen and glutamine synthetase, indicating TGFβ pathway activation in proliferating Müller glia. Inhibiting the TGFβ signaling pathway using a small molecule inhibitor (SB431542) resulted in accelerated recovery from retinal degeneration. Accordingly, we observed increased cell proliferation in the outer nuclear layer at days 3 to 8 after MNU treatment. In contrast to the observations in the heart and the fin, the inhibition of the TGFβ signaling pathway resulted in increased proliferation after the induction of retinal degeneration. A better understanding of the underlying pathways with the possibility to boost retinal regeneration in adult zebrafish may potentially help to stimulate such proliferation also in other species.

## Introduction

Zebrafish (Danio rerio) is an important model system in visual research, amongst others, as its retina shows the typical structure of vertebrates and is rich in cone photoreceptors [1–6]. The lifelong retinal neurogenesis in zebrafish is particularly interesting [7]. Under physiological conditions, the ciliary marginal zone (CMZ) and rod progenitors in the outer nuclear layer (ONL) maintain stable rod photoreceptor density in a continuously growing eye [7–12]. Furthermore, the zebrafish retina regenerates even after severe damage [13–16]. Thereby, proliferating de-differentiated Müller glia exhibit the ability to replace all types of neurons to reconstitute the damaged retina, forming also rod progenitors that regenerate photoreceptor cells [7,15,17–20].

We have recently introduced the N-methyl-N-nitrosourea (MNU)-induced chemical model of rod photoreceptor degeneration in zebrafish [14,21]. This model is ideal for analyzing the signaling pathways involved in retinal regeneration, as it selectively damages photoreceptors [14]. MNU acts similarly in various species [22–24]. Therefore, this model provides a method to elucidate why regeneration occurs in adult zebrafish but not in mammals.

Previously, the transforming growth factor beta (TGFβ) pathway was identified to play a crucial role in the regeneration of the heart [25] and the fin [26] in adult zebrafish. TGFβ is among the most important ligands involved in cell behavior because it modulates cell migration, proliferation and death during development and tissue repair [27]. TGFβ enhances extracellular matrix production after injury [27–29]. The hyper-activation of the TGFβ pathway may lead to a fibrotic response [27]. Significantly altered infarct tissue and impaired heart regeneration were observed after the inhibition of this pathway using the chemical inhibitor SB431542 [25], which is a potent and specific inhibitor of the TGFβ/activin-dependent pathway.

TGFβ belongs to the TGFβ superfamily, which also includes the activins. The binding specificity of this superfamily is achieved via the combination of type I and type II receptors. The small molecule inhibitor SB431542 blocks the corresponding TGFβ type I receptors of TGFβ and the activins via the activin receptor-like kinases (ALK) 4, 5 and 7. Blocking ALK 4 and 5 hinders the phosphorylation and, therefore, the activation of Smad2/3 [30]. Therefore, in our study both TGFβ and activin signaling is blocked. For simplicity, we are referring to this as "blocking the TGFβ pathway".

A recent study showed that retinal regeneration in adult zebrafish requires the regulation of TGFβ signaling by the co-repressors TGif1 and Six3b [31]. The functional disruption of these co-repressors resulted in a significant reduction in photoreceptor regeneration [31]. Their results indicate that Smad2/3-mediated TGFβ signaling acts to inhibit proliferation of neuronal progenitors following photoreceptor destruction in the adult zebrafish retina [31]. However, the outcome of TGFβ pathway inhibition remains unclear. To resolve this issue, we blocked this pathway using the specific inhibitor SB431542 during retinal regeneration after MNU-induced photoreceptor degeneration.

## Materials and Methods

### Animals

Wild-type zebrafish (Danio rerio) of the AB (Oregon) strain aged from 9 to 12 months were used. The fish were maintained under standard conditions [32,33] in water at a temperature of approximately 26.5°Celsius and were raised in a 14-hour light/10-hour dark cycle. The experimental research on animals was approved by the Cantonal Veterinary Office of Fribourg (Switzerland) and adhered to the Association for Research in Vision and Ophthalmology (ARVO) Statement for the Use of Animals in Ophthalmic and Vision Research.

### MNU treatment and inhibition protocol

The fish were randomly assigned to either the uninhibited or the inhibited group. In the latter group, the TGFβ/activin pathway was blocked using the small molecule inhibitor SB431542 (Tocris, Bristol, UK). The inhibitor was dissolved in dimethyl sulfoxide (DMSO) and added to the water of the fish tank, beginning one day prior to the induction of retinal degeneration, and was refreshed every third day. The final concentration in the water of the fish tank was 20 μM SB431542 and 0.1% DMSO. The uninhibited fish were held in water with 0.1% DMSO. Retinal degeneration was induced in both groups by placing the zebrafish in water containing 150 mg/l N-methyl-N-nitrosourea (MNU, Sigma, St. Louis, MO, USA) for one hour as previously described by our group [14].

### Histology and cell quantification

Histology was performed before (day 0; uninjured) or at 1, 3, 5, 8, 15 or 30 days after MNU treatment. To verify that the cell count was higher in the inhibited group than in the uninhibited group (see results), the experiment was repeated twice for day 8. After euthanasia with tricaine methanesulfonate 0.3 mg/ml (Sigma-Aldrich, Buchs, Switzerland), the eyes were enucleated, fixed using 4% paraformaldehyde, and embedded in paraffin. Then, 5-μm sections were sliced as previously described [14]. The sections were stained with hematoxylin and eosin (H&E). Images were captured with a Nikon Eclipse 80i microscope and were globally adjusted for white balance and brightness with Adobe Photoshop. Sagittally oriented central sections at the level of the optic nerve head were used for the measurements. The number of cells in the inner nuclear layer (INL) and the outer nuclear layer (ONL) was manually determined at the same position in the mid-periphery on both sides of the eye (the size of the counted area corresponded to a retinal section of 100 μm in length).

### TUNEL staining and immunohistochemistry

Paraffinized tissue sections were also used for TUNEL staining (In Situ Cell Detection Kit, Fluorescein; Roche Applied Science, Rotkreuz, Switzerland) and immunohistochemistry [14]. The following primary antibodies were used: mouse anti-proliferating cell nuclear antigen (PCNA) to detect cell proliferation (1:200 dilution; Abcam, Cambridge, UK), mouse anti-glutamine synthetase (GS) to detect Müller glia (1:200; Millipore, Billerica, MA, USA) and rabbit anti-phosphorylated Smad3 (P-Smad3) to assess TGFβ pathway activity (1:50; ab52903, Abcam, Cambridge, UK). Goat anti-rabbit and anti-mouse secondary antibodies conjugated to Alexa 488 nm or 594 nm, respectively (1:500; Life Technologies, Paisley, UK), were utilized. Immunohistochemistry for P-Smad3 was performed for all time-points (0, 1, 3, 5, 8, 15 or 30 days after MNU treatment). Double-staining was performed for P-Smad3 and PCNA or GS using the above antibodies. Apoptosis (TUNEL-positive cells) and cell proliferation (PCNA-positive cells) were assessed by counting the cells as described above for cell counting in the H&E-stained sections. The size of the counted area corresponded to a retinal length of 500 μm (TUNEL-positive cells) or 180 μm (PCNA-positive cells).

### In situ hybridization

After deparaffinization, in situ hybridizations were performed as described by Chablais and Jazwinska at days 0, 1, 5, and 30 after the induction of retinal degeneration [34]. The primers are listed in the supplementary (**S1 Table**).

### Statistical analysis

Statistical analysis was performed using GraphPad software (version 6.0f, GraphPad Software, La Jolla, CA, USA). Intergroup comparisons were performed via one-way analysis of variance (ANOVA) followed by the Bonferroni multiple comparison post hoc test. The level of significance was set at a P value of 0.05. Cell counts were performed on 3 eyes from 3 zebrafish for each time point. The experiments were repeated twice for day 0 and day 8 (H&E staining only) to verify the observed increased cell counts in the inhibited group. For each eye, the cells in two corresponding areas (opposite sides of the optic nerve) were counted, and the mean values were calculated.

## Results

**Fig 1:**
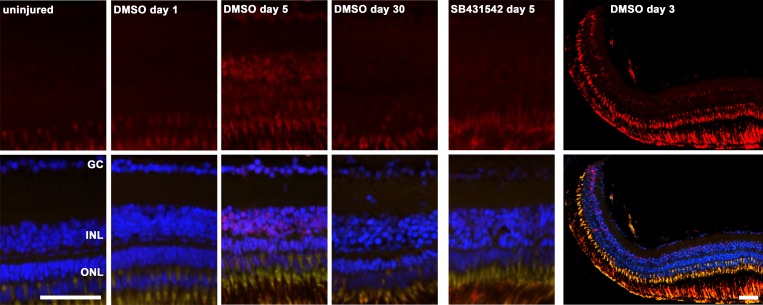
Immunohistological staining for P-Smad3 as an indicator of TGFβ pathway activation. The red channel with P-Smad3 staining is shown in the figures above, whereas overlay with the green (autofluorescence of photoreceptor outer segments) and blue channel (DAPI) is shown below. No relevant staining for P-Smad3 (red) was observed in the uninjured retina and one day after induction of retina degeneration with MNU. Starting at day 3 and until day 8, immunohistochemical staining for P-Smad3 revealed the activation of the TGFβ pathway (exemplarily, day 5 is shown). At day 15 and thereafter, no relevant activation was observed anymore (exemplarily, day 30 is shown). When the TGFβ pathway was inhibited (small molecule inhibitor SB431542), reduced staining for P-Smad3 was observed, when compared to the non-inhibited group in 0.1% dimethyl sulfoxide (DMSO). Lower magnification of retina 3 days after MNU treatment, including the peripheral retina is shown on the right. Cell nuclei are stained with DAPI (blue). The scale bar indicates 50 μm. GC: ganglion cells; INL: inner nuclear layer; ONL: outer nuclear layer.

After inducing retinal degeneration using 150 mg/l MNU, maximal activation of the TGFβ pathway occurred between days 3 and 8 as demonstrated by immunohistochemistry for phosphorylated Smad3 (P-Smad3) (**Fig 1**; representative immunohistochemistry for day 5 is shown). TGFβ pathway activation was primarily observed in the INL and to some degree in the ONL at the late time points (days 5 and 8). P-Smad3 staining is observed from the central to the peripheral retina, often more pronounced in the peripheral retina towards the ciliary marginal zone (**Fig 1**). Consistent with the inhibition of the TGFβ receptor by SB431542, partly reduced Smad3 activation was observed in the inhibited group. For both groups, no relevant P-Smad3 was observed at baseline (day 0; uninjured retina), day 1 and between days 15 and 30 after induction of retina degeneration (**Fig 1**, exemplarily, uninjured retina, day 1, 5 and 30 are shown).

**Fig 2:**
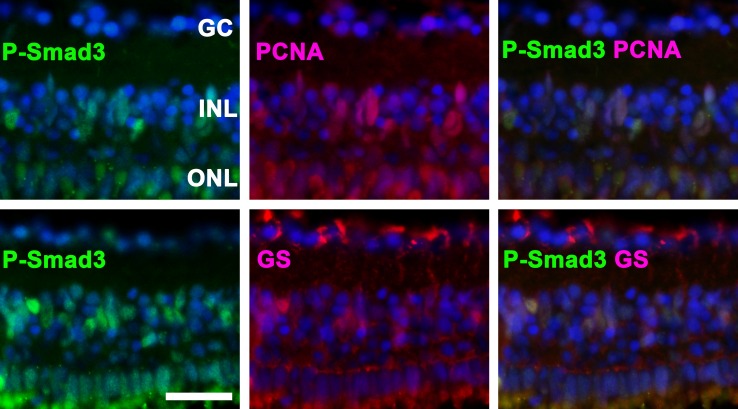
P-Smad3 is activated in proliferating cells. Top: The co-localization of P-Smad3 and proliferating cell nuclear antigen (PCNA) indicates that Smad3 is activated in proliferating cells. Bottom: P-Smad3-positive cells in the inner nuclear layer (INL) co-localized with glutamine synthetase (GS), suggesting that these cells are Müller glia. Representative immunohistochemical staining at day 3 is depicted. Cell nuclei are stained with DAPI (blue). The scale bar indicates 25 μm. GC: ganglion cells, ONL: outer nuclear layer.

Based on immunohistochemistry, double-staining for P-Smad3 and PCNA (a proliferation marker) or GS (a Müller glial marker) revealed the co-localization of these proteins, suggesting that the TGFβ pathway is activated in proliferating Müller glia (**Fig 2**).

**Fig 3:**
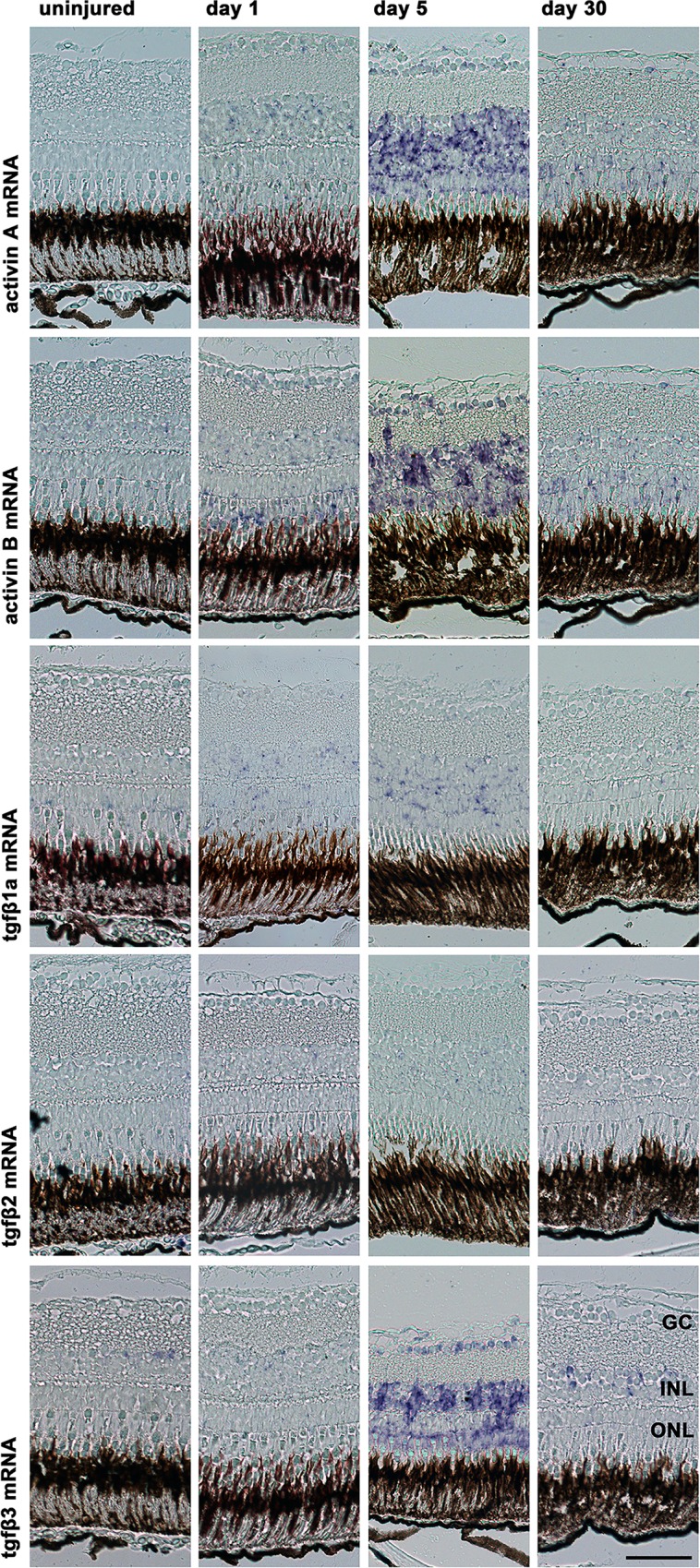
In situ hybridization with activin A and B as well as tgfβ1a, 2 and 3 antisense probes in zebrafish after the induction of retinal degeneration by MNU. Expression of these genes was detected beginning at day 1 and peaking at day 5. The highest staining intensity was observed for tgfβ3 and activins A and B, whereas only modest staining was observed for tgfβ1a and 2. These ligands were primarily detected in the inner nuclear layer (INL). The scale bar indicates 50 μm. GC: ganglion cells, ONL: outer nuclear layer.

In situ hybridization for activin A and B and for TGFβ1a, 2 and 3 showed expression of these genes as soon as one day after the induction of retinal degeneration. On day 5, the expression increased further, whereas it nearly returned to baseline on day 30 (**Fig 3**). The highest staining intensity was observed for TGFβ3, activin A and activin B, whereas only modest staining was observed for TGFβ1a and only minimal staining for TGFβ2. At day 5, the expression of these mRNAs is mainly in the INL, where the pattern corresponds to the distribution and morphology of Müller glia cells. At day 30, the (weak) expression of activin A and B is mainly in the ONL.

**Fig 4:**
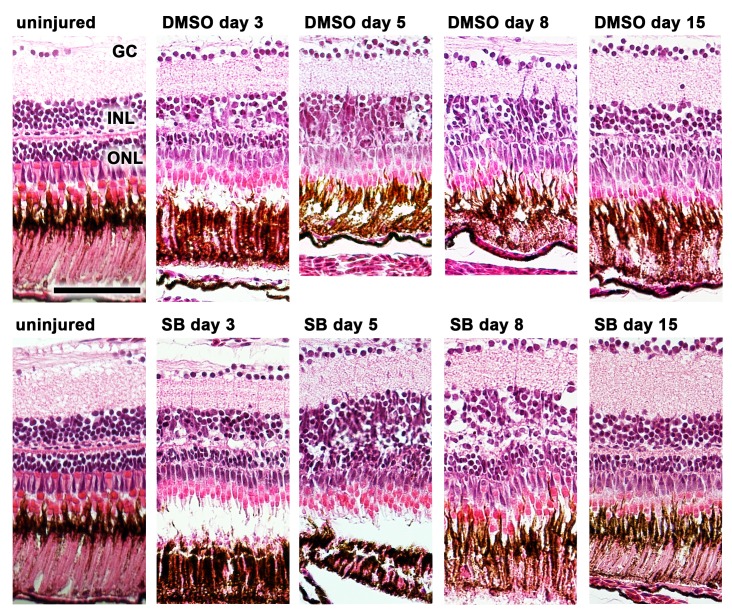
H&E staining of zebrafish retinas before (uninjured) and after induction of retina degeneration with MNU. In the non-inhibited (0.1% dimethyl sulfide, DMSO) and inhibited group (small molecule inhibitor SB431542), a reduction of rod cells was observed starting at day 3. In the non-inhibited group the reduction of rod photoreceptors persisted until day 8, whereas in the group with the inhibited TGFβ pathway (small molecule inhibitor SB431542) a rapid recovery was observed already at day 5. Scale bar indicates 50 μm. GC: ganglion cells, INL: inner nuclear layer, ONL: outer nuclear layer, SB: SB431542

**Fig 5:**
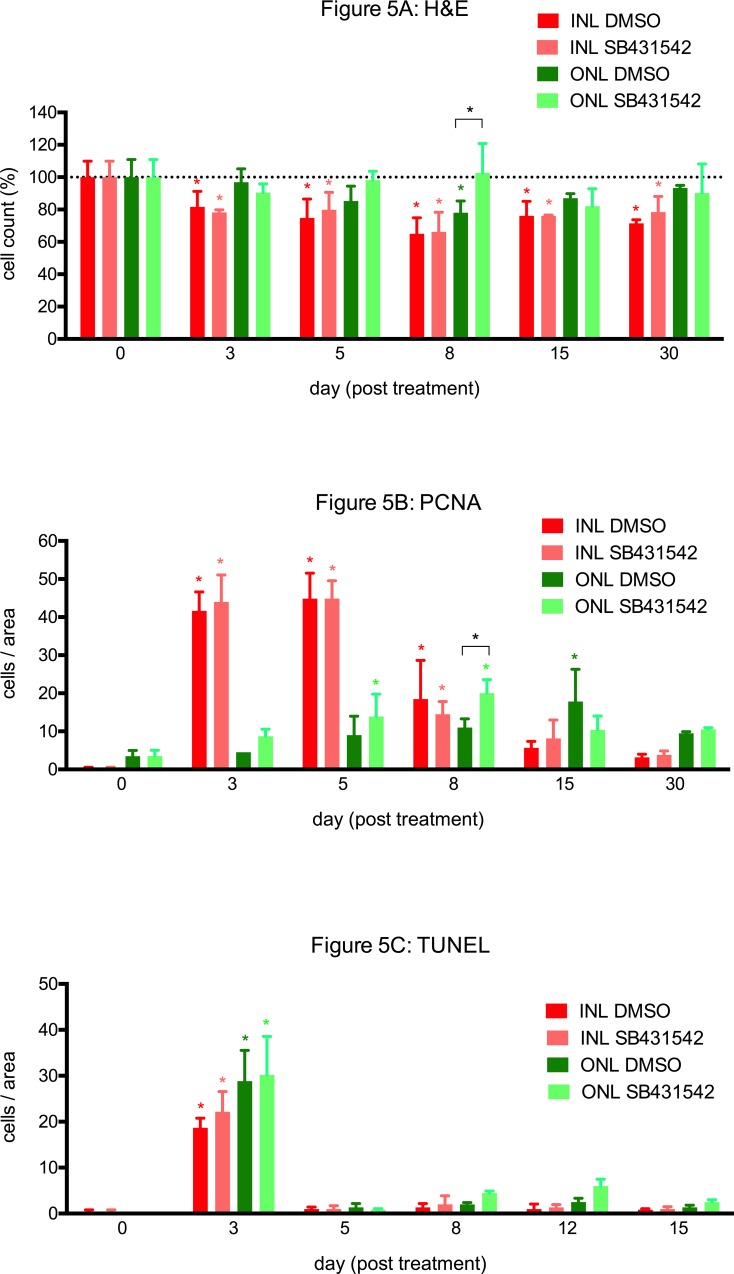
Cell quantifications of H&E, PCNA and TUNEL stainings. **A.** After the induction of retinal degeneration, cell loss in the outer nuclear layer (ONL) was observed in H&E stained paraffin sections. In zebrafish in which TGFβ signaling was inhibited (ONL SB431542), the cell count in the ONL was slightly decreased at day 3 compared to the baseline level (uninjured retina; day 0) but rapidly returned to it thereafter. Alternatively, in the uninhibited group (ONL DMSO), the cell count remained reduced until day 8. Compared to the baseline level, the number of cells of the INL of both groups (INL DMSO and INL SB431542) was significantly reduced beginning at day 3, and this reduction persisted up to day 30. **B.** After the induction of retinal degeneration using MNU, a significant increase in PCNA-positive cells, indicating proliferation, was observed in the INL (INL DMSO and INL SB431542) between days 3 and 8 (peaking at days 3 and 5). Whereas the maximal increase in PCNA-positive cells in the ONL was observed at days 5 and 8 in the TGFβ-inhibited group (ONL SB431542), this increase was the highest at day 15 in the uninhibited group (ONL DMSO). Overall, the inhibited group exhibited significantly more PCNA-positive cells than the uninhibited group (p<0.05 at day 8). **C.** The most TUNEL-positive cells, indicating apoptosis, were observed at day 3 in the ONL and the INL of both the TGFβ-inhibited (SB431542) and uninhibited groups (DMSO). In addition, some TUNEL-positive cells were observed at days 8 and 12 in the ONL of the TGFβ-inhibited group (ONL SB431542). The asterisks (*) indicate a significant difference (p<0.05) compared to baseline, and the asterisks with squared brackets indicate a significant difference (p<0.05) between inhibited and uninhibited TGFβ signaling.

Cell quantification was performed on H&E sections (**Figs 4 and 5A)**. Different changes in the cell counts in the ONL were observed between the inhibited and uninhibited groups. In zebrafish in which TGFβ signaling was inhibited, the number of cells in the ONL was slightly decreased on day 3 but subsequently exhibited a rapid increase to the baseline values on day 5, peaking on day 8 (p<0.0001 compared to the uninhibited group). In comparison, the cell count in the ONL of the uninhibited group was lower than in the inhibited group and approached the baseline values not before day 30. To verify this difference, the experiment was independently repeated twice for day 8. A similar decrease in the number of cells in the INL compared to the baseline values was observed for both groups beginning on day 3; this decrease reached a minimum on day 8 and persisted up to day 30.

**Fig 6:**
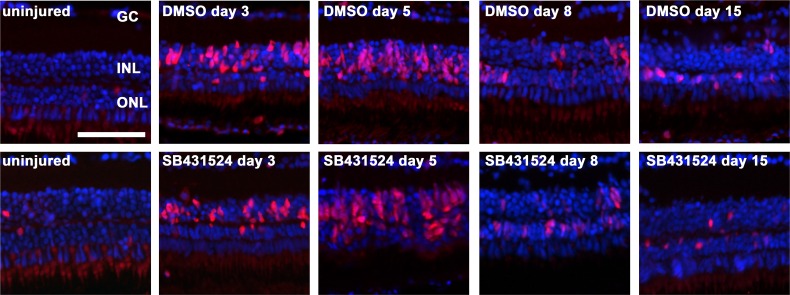
Cell proliferation in the zebrafish retina exposed to 150 mg/l MNU. Proliferating cell nuclear antigen (PCNA) positive cells (red) indicate proliferation. Cell proliferation in the inner nuclear layer (INL) was highest at day 3 and 5, with no relevant difference between the non-inhibited (0.1% dimethyl sulfide, DMSO) and inhibited group (small molecule inhibitor SB431542). In contrast, proliferation in the outer nuclear layer (ONL) was higher in the inhibited group between 3 and 8. Cell nuclei are stained with DAPI (blue). Scale bar indicates 50 μm. GC: ganglion cells.

No significant difference in cell proliferation in the INL, which peaked between days 3 and 8, was observed between TGFβ inhibition and non-inhibition (p>0.05 at each time point; **Fig 5B**). However, cell proliferation in the ONL was distinct between the two groups. The maximal number of proliferating cells in the ONL was observed on days 5 and 8 in the inhibited group but on day 15 in the uninhibited group (**Fig 5B**). Furthermore, the total number of PCNA-positive cells was higher in the inhibited group than in the uninhibited group (p<0.05 at day 8; **Figs 5B and 6**).

**Fig 7:**
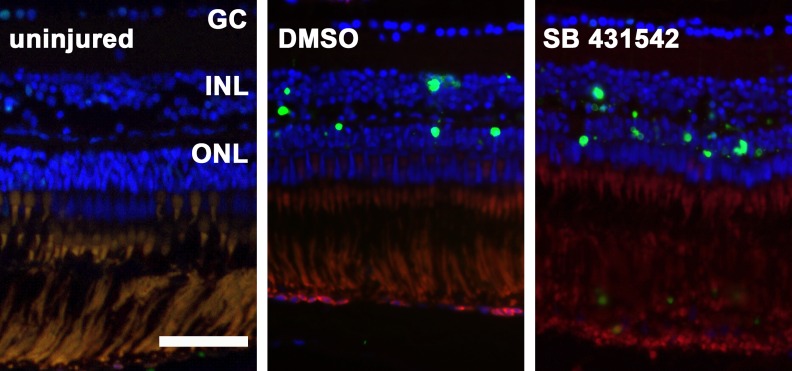
TUNEL positive cells in the zebrafish retina after exposure to MNU. In uninjured zebrafish retina there are merely no TUNEL positive cells. Three days after exposure to 150 mg/l MNU, both the non-inhibited (0.1% dimethyl sulfide, DMSO) and the inhibited group (small molecule inhibitor SB431542) show a considerable amount of TUNEL positive cells (green) in the outer nuclear layer (ONL) and to a lesser degree in the inner nuclear layer (INL). Cell nuclei are stained with DAPI (blue). Scale bar indicates 50 μm. GC: ganglion cells.

In zebrafish in which TGFβ signaling was either inhibited or uninhibited, TUNEL staining indicated a peak of apoptosis on day 3 (p<0.0001 for each), which was primarily detected in the ONL (**Figs 5C and 7**). Furthermore, the inhibited group displayed some TUNEL-positive cells in the ONL on days 8 and 12. This result agrees with the decrease in the cell number between days 8 and 15.

## Discussion

Teleost fish, such as zebrafish, exhibit the potential to regenerate most of their organs [14,16,25,26]. After injury to the zebrafish retina, Müller glia cells are able to regenerate all types of retinal neurons [35–39]. Furthermore, Müller glia replenish the pool of rod photoreceptor progenitor cells in the ONL [15,16,18,19,40,41]. In this study we have used a retinal degeneration model using MNU, as previously described by our group [14]. Compared to our previous study [14], a lower amount of rod degeneration and an earlier start of proliferation were observed in the present study. One explanation may be that we now have used younger zebrafish (age 9–12 months), whereas in the other study fish were older (age 12–24 months). Especially in the inhibited group, rod photoreceptor cells did not decrease substantially, although there was a similar increase in TUNEL positive cells as in the non-inhibited group. Therefore the possibility that the inhibitor protects rods from degeneration is unlikely and the missing cell loss explained by the stronger proliferation.

Despite significant scientific efforts, the reason why this regeneration occurs in some species but not in others remains unclear. In the present study, we aimed to elucidate the role of the TGFβ signaling pathway during retinal regeneration. Recent studies have shown that the activation of the TGFβ signaling pathway is necessary for the regeneration of the zebrafish heart and fin [25,26]. Müller glia express TGFβ receptors, and serve as a source of TGFβ [31,42–44] and thrombospondin-1 [45], an activator of TGFβ [46]. These findings concur with our in situ experiments, in which we observed increased expression of TGFβ and the related activins A and B in the INL during retinal regeneration. In our study we observed that immunohistochemical staining for P-Smad3 co-localized with that for PCNA and GS. This result indicated that the TGFβ pathway is activated in dividing Müller glia. Interestingly, at day 30 the remaining expression of activin A and B is relatively higher in the ONL than in the INL. This is in line with the observation that at that time point more PCNA positive cells are found in the ONL than in the INL. Furthermore, when the TGFβ pathway is inhibited, there are more PCNA positive cells in the ONL between day 3 and 8, significantly so at day 8. These two observations may indicate that the TGFβ pathway is also activated in rod progenitors in the ONL.

Lenkowski et al. reported that the overall pattern of transcriptional changes in members of the Smad2/3 signaling pathway suggests that TGFβ signaling initially is upregulated in the Müller glia after light lesion, but then is quickly suppressed. They concluded that down regulation of Smad2/3 signaling in the Müller glia is particularly important for the proliferative, neurogenic, response of Müller glia to light-induced destruction of photoreceptors in the adult zebrafish [31]. This is in line with our study, where we have found increased proliferation when the TGFβ signaling pathway is inhibited. Furthermore, in our study we observed elevated P-Smad3 levels between day 3 and 8. Taken together the results of the study of Lenkowski et al. and ours, it may be speculated that the initial inhibition of TGFβ is important for proliferation, and thereafter TGFβ signaling promotes differentiation.

Our study reveals that inhibiting the TGFβ signaling pathways leads to accelerated recovery from retinal degeneration, including increased cell proliferation in the ONL at days 3 to 8 after MNU treatment. These findings are remarkable as the TGFβ pathway was shown to be crucial for regeneration in the fin and the heart [25,26]. However, our findings are in line with the observations of Lenkowski et al. [31], who described that increased activation of the TGFβ pathway (via the functional disruption of the co-repressors Tgif1 and Six3b) hampers retinal regeneration. Interestingly, the inhibition of Smad signaling using the same small molecule inhibitor (SB431542) leads to the rapid and complete neural conversion of human embryonic stem cells [47]. In vitro experiments by Close et al. revealed that aged rat Müller glia inhibited the proliferation of retinal progenitors and Müller glia [48]. As proliferation was restored when TGFβ signaling was inhibited, the authors hypothesized that TGFβ signaling maintains mitotic quiescence in the postnatal rat retina [48]. Furthermore, activin A (a member of the TGFβ superfamily that also signals via P-Smad2/3) has been shown to promote the differentiation of progenitors into photoreceptors in rodent retinal cell cultures [49].

The opposing effects of the TGFβ signaling pathway on different organs (heart and fin vs. eye) during regeneration in the same species is fascinating. This difference may be explained by the variety of biological effects of TGFβ and the interaction between the TGFβ pathway and other signaling pathways, as nicely summarized by Lenkowski et al. for the retina [31]. In zebrafish, TGFβ signaling-induced scarring is crucial for heart regeneration, and TGFβ signaling-induced extracellular matrix deposition is required for fin regeneration [25,26]. In neuronal tissue, including the eye, glial scarring is associated with the inhibition of cell proliferation. Indeed, in mammals, glial scarring after retinal damage (e.g., retinal detachment) is thought to be disadvantageous [50–53].

Our approach reveals a new way to stimulate retinal regeneration, at least in zebrafish. Complementary to the findings of Lenkowski et al. [31], who stated that Smad2/3-mediated TGFβ signaling inhibits proliferation of neuronal progenitors, we observed increased retinal regeneration when the TGFβ pathway was inhibited. Whether such an effect may also be achieved in mammals is unknown, but previous in vitro studies indicating similar effects of TGFβ signaling modulation on Müller glia proliferation in mammals are encouraging [47–49]. Retinal injury or degeneration is an important cause of visual impairment or blindness; therefore, an improved understanding of mechanisms that enhance retinal regeneration would be highly desirable. In the long term, this understanding may also provide insight into potential treatments for degenerative retinal diseases. We hope that our current findings contribute to such evidence.

## Supporting Information

S1 Appendix Dataset for cell counting. (XLSX)

S1 Table Primers for the PCR amplification of genes to generate antisense probes for in situ hybridization. (DOCX)
