# Supplementary material for: Inhibition of the TGFβ Pathway Enhances Retinal Regeneration in Adult Zebrafish
Source: PLoS One. 2016 Nov 23;11(11):e0167073. doi: 10.1371/journal.pone.0167073 (PMC5120850; doi:10.1371/journal.pone.0167073)
Supplement: S1 Table — (DOCX) [file pone.0167073.s002.docx]

**S1 Table. Primers for the PCR amplification of genes to generate antisense probes for in situ hybridization.** To generate antisense probes, the reverse primers were synthesized via the addition of the T3 polymerase promoter.

| Gene | Gene ID | Forward primer | Reverse primer | Prod. (bp) |
| --- | --- | --- | --- | --- |
| tgfβ1a | NM_182873 | TGCAGGATGAGGATGAGGAC | CTGTGTACCCGCAATCCTTG | 780 |
| tgfβ2 | NM_194385 | ACGCCAAAGAAGTGCACAAG | CTGTCCGTATCTGTGGAGCG | 762 |
| tgfβ3 | NM_194386 | AACCTGAGCACCTCCAGGAC | GCTGCACTTGCAGGATTTG | 765 |
| activin A | NM_130916 | GGGATCCTCCTGCTGCTAAT | AGTGGAAGGACAGCGAGTTG | 991 |
| activin B | NM_131068 | AAGATTCGGGGAGAATGGAC | GTTAGGCACGTCACGTTTGA | 1004 |
